# Driving and attention deficit hyperactivity disorder

**DOI:** 10.1007/s00702-015-1465-6

**Published:** 2015-09-29

**Authors:** Anselm B. M. Fuermaier, Lara Tucha, Ben Lewis Evans, Janneke Koerts, Dick de Waard, Karel Brookhuis, Steffen Aschenbrenner, Johannes Thome, Klaus W. Lange, Oliver Tucha

**Affiliations:** 1 Department of Clinical and Developmental Neuropsychology, University of Groningen, Grote Kruisstraat 2/1, 9712 TS Groningen, The Netherlands; 2 Traffic and Environmental Psychology Group, University of Groningen, Groningen, The Netherlands; 3 Department of Clinical Psychology and Neuropsychology, SRH Clinic Karlsbad-Langensteinbach, Karlsbad-Langensteinbach, Germany; 4 Department of Psychiatry and Psychotherapy, University of Rostock, Rostock, Germany; 5 Department of Experimental Psychology, University of Regensburg, Regensburg, Germany

**Keywords:** ADHD, Adulthood, Driving, Traffic, Mobility, Accidents, Speeding

## Abstract

Adults with attention deficit hyperactivity disorder (ADHD) suffer from various impairments of cognitive, emotional and social functioning, which can have considerable consequences for many areas of daily living. One of those areas is driving a vehicle. Driving is an important activity of everyday life and requires an efficient interplay between multiple cognitive, perceptual, and motor skills. In the present study, a selective review of the literature on driving-related difficulties associated with ADHD is performed, seeking to answer whether individuals with ADHD show increased levels of unsafe driving behaviours, which cognitive (dys)functions of individuals with ADHD are related to driving difficulty, and whether pharmacological treatment significantly improves the driving behaviour of individuals with ADHD. The available research provides convincing evidence that individuals with ADHD have different and more adverse driving outcomes than individuals without the condition. However, it appears that not all individuals with ADHD are affected uniformly. Despite various cognitive functions being related with driving difficulties, these functions do not appear helpful in detecting high risk drivers with ADHD, nor in predicting driving outcomes in individuals with ADHD, since impairments in these functions are defining criteria for the diagnoses of ADHD (e.g., inattention and impulsivity). Pharmacological treatment of ADHD, in particular stimulant drug treatment, appears to be beneficial to the driving difficulties experienced by individuals with ADHD. However, additional research is needed, in particular further studies that address the numerous methodological weaknesses of many of the previous studies.

## Introduction

Driving is an important activity of daily living, which can be crucial for independent living, social integration, quality of life, life-satisfaction, and even mental health, such as susceptibility to depression (e.g., Man-Son-Hing et al. 2007; Novack et al. 2010). However, driving also represents a rather complex task requiring the dynamic, mostly automatic, and hopefully error-free interplay of various perceptive, motor and cognitive functions. With regard to cognition, the integrity of attentional processing, impulse control, and executive functions appear to be particularly relevant for safe driving; however, other functions such as memory or visuospatial functions are also required (Lansdown 2002; Lincoln and Radford 2013; Rizzo and Kellison 2010). A driver’s emotional state and personality may also play an important role in safe driving. If this interplay of functions is disrupted, drivers may place themselves as well as other individuals at considerable risk of being involved in road traffic accidents. According to the WHO, road traffic accidents are the 8th leading cause of death globally with more than 1.2 million people killed in 2010 and for individuals aged 15–29, road traffic accidents even represent the 1st leading cause of death worldwide (World Health Organization 2013). The costs of road crash injuries have been estimated to be above €180 billion for the EU per year (Peden et al. 2004).

Inattention, impulsivity and executive dysfunctioning are assumed to represent the core cognitive symptoms of attention deficit hyperactivity disorder (ADHD). This assumption is supported by a variety of studies showing that adults with ADHD display impairments of selective attention (Corbett and Stanczak 1999; Dinn et al. 2001; Lovejoy et al. 1999), divided attention (Jenkins et al. 1998; Tucha et al. 2008; Woods et al. 2002), flexibility/set shifting (Hollingsworth et al. 2001; Rohlf et al. 2012; Tucha et al. 2006b) and vigilance/sustained attention (Epstein et al. 2001; Tucha et al. 2009; Weyandt et al. 1998). The most robust findings concerning attention have been reported for impairments of selective attention and vigilance/sustained attention, with differences of medium effect size between adults with ADHD and healthy controls (Boonstra et al. 2005; Hervey et al. 2004; Schoechlin and Engel 2005; Thome et al. 2012). The most robust finding regarding executive functions is that individuals with ADHD have an impaired working memory (Alderson et al. 2013; Dowson et al. 2004); however, impairments of impulse control (Chamberlain et al. 2007; Hervey et al. 2004; Ossmann and Mulligan 2003), fluency functions (Boonstra et al. 2005; Dinn et al. 2001; Tucha et al. 2005), planning and problem solving (McLeod et al. 2004; Schoechlin and Engel 2005; Tucha et al. 2011a), concept formation (Antshel et al. 2010; Horton 1996), cognitive flexibility (Boonstra et al. 2010; Halleland et al. 2012), social cognition (Ibanez et al. 2011), time perception (Barkley et al. 2001), and decision making behaviour (Agay et al. 2010; Groen et al. 2013) have also been repeatedly reported.

Because of these impairments, individuals with ADHD may have a higher likelihood to cause or, at least, be involved in traffic accidents. For example, drivers with ADHD may have difficulty regulating their attention between driving and other tasks (e.g., listening to the radio, looking at objects outside traffic, or having a conversation), may pay less attention to the road, and consequently miss important environmental cues or the behaviours of other road users. Individuals with ADHD may also be more easily distracted and, therefore, may not anticipate behaviour of others or show delayed reactions to, for example, other road users who might lose control over their vehicles. Beside their cognitive impairments, adults with ADHD might also be more prone to be involved in road traffic accidents because of their emotional-motivational dysfunctions and personality traits (Conzelmann et al. 2011; Skirrow and Asherson 2013). The resemblance of the clinical presentation of adults with moderate to severe ADHD and descriptions of aggressive and many accident-involved drivers is remarkable. The latter have been described as predominantly male, single, thrill-seeking, impulsive, irresponsible, aggressive, poorly adjusted with a history of a difficult childhood, emotional unstable with increased levels of stress at home and work, delinquent, and often have a history of abuse of alcohol or other drugs (Beirness 1993; Dahlen et al. 2005; Deery and Fildes 1999; DiFranza et al. 1986; Holzapfel 1995; Jonah 1997; Lincoln and Radford 2013; Rizzo and Kellison 2010; Schwebel et al. 2006; Suchman 1970).

Because of these relations between ADHD and the characteristics associated with poor/risky driving, as well as the patients’ reports about driving difficulties during their clinical assessments, a number of creative and work-intensive studies have been performed to assess driving-related risks and impairments associated with ADHD in adolescents and adults suffering from the condition. In the present article, we give an overview of these studies and seek to answer whether individuals with ADHD have shown increased levels of unsafe driving behaviour, what cognitive (dys)functions of individuals with ADHD are related to unsafe driving and whether pharmacological treatment improves the driving behaviour of adolescents and adults with ADHD significantly. The answers to these questions are of high relevance for patients, health specialists, and society alike.

## Methods

A selective literature review was carried out to identify studies on driving-related difficulties of adults with ADHD. A thorough literature search was performed using electronic data bases PsycINFO, PubMed, and Web of Science including available literature up until September 2015. Initial search terms that were used were ‘ADHD’, ‘ADD’, ‘attention deficit hyperactivity disorder’ or ‘attention deficit disorder’ in combination with ‘driving’, ‘driver’, ‘drive’, ‘car’, ‘road’, ‘traffic’, ‘speeding’, ‘accident’, or ‘collision’. The literature was considered for inclusion if the study addressed one of the research questions, i.e., (1) whether individuals with ADHD show increased levels of unsafe driving behaviours, (2) which cognitive (dys)functions of individuals with ADHD are related to driving difficulty, and (3) whether pharmacological treatment significantly improves the driving behaviour of individuals with ADHD. The reference lists of the initial studies were then used to locate other relevant studies. Furthermore, studies were only included if they were written in English, were peer reviewed, included a sample of adults formally diagnosed with ADHD, and investigated driving behaviour by means of either self-reports, informant reports, official reports, driving simulators or on-road driving tests.

## Results and discussion

### Do individuals with ADHD show increased levels of unsafe driving behaviour?

Different approaches were applied in the assessment of the driving performance and behaviour of individuals with ADHD. These approaches comprise self-, informant- and official reports, driving simulator studies, and on-road driving assessments. All these approaches have their strengths and weaknesses, which are important to consider when interpreting the study results. Therefore, the available findings will subsequently be presented for each of these methods separately and then summarised in a brief synopsis.

#### Self-, informant- and official reports

A number of studies have collected data on driving performance and driving behaviour of adolescents and adults with ADHD by analysing patients’ self-reports, informants’ reports (e.g., parents) and/or official driving records (e.g. Aduen et al. 2015; Barkley et al. 1993, 1996, 2002; Beck et al. 1996; Biederman et al. 2006; Chang et al. 2014; Cox et al. 2000, 2011a; Fischer et al. 2007; Fried et al. 2006; Garner et al. 2012; Jerome et al. 2006; Koisaari et al., 2015; Murphy and Barkley 1996; NadaRaja et al. 1997; Narad et al. 2015; Richards et al. 2002, 2006; Rosenbloom and Wultz 2011; Safiri et al. 2013; Sobanski et al. 2008; Swenson et al. 2004; Thompson et al. 2007; Weiss et al. 1979; Woodward et al. 2000). The main advantage of self- and informant-reports is that they are cheap and easy to apply. Self-reports also reflect patients’ reported perception of their experiences, which makes self-reports a unique and crucial source of information. In contrast, official records represent a more objective measure, are free of referral bias, and allow the assessment of considerable sample sizes (Chang et al. 2014). The results of studies analysing self-, informant- and official reports demonstrate that individuals with ADHD make considerably more frequent, but also more serious, driving errors than healthy individuals with equivalent driving experience. Adolescents and adults with ADHD reported significantly higher numbers of traffic citations, and rates of driving without licence, as well as more involvement in traffic accidents, than non-ADHD suffering drivers (NadaRaja et al. 1997; Reimer et al. 2005; Richards et al. 2006; Thompson et al. 2007). Moreover, individuals with ADHD show higher rates of collisions on highways, rear-ended accidents, and have their licence suspended or revoked more often (Fischer et al. 2007; Fried et al. 2006). These findings are supported by parents’ reports and further corroborated by official driving records. Table 1 provides a list of adverse driving outcomes of individuals with ADHD as compared to those who do not have ADHD.

**Table 1:** Adverse driving outcomes found in individuals with ADHD in comparison to those who do not have ADHD as assessed via various methods

Self-report, informant-report or official driving records^a^	Driving simulation^b^	On-road testing^c^
More traffic accidents	More collisions and crashes	More collisions and crashes
More accidents on highways	More speeding	
		More speeding
	More scrapes	
		More driving errors
	Poorer steering control	
More rear-ended accidents while driving		More hard braking
	Increased lane swerving	
		More sudden deceleration More weaving of the car
More hit-and-run accidents	Slower and more variable reaction times	
More fatal accidents		
	More driving errors	
More often the subject at fault in an accident		
	Impaired driving score	
More traffic citations (e.g., for speeding and other traffic violations)	More often fatigued	
More convictions and arrests		
More licence suspensions		
More reckless and risky driving		
More often driving without licence		
More often driving illegally before licensed to drive		
More parent-reported driving problems		
More driving-related anger and aggression		
More often driving under the influence of alcohol		

^a^Aduen et al. (2015); Based on Barkley et al. (1993, 1996, 2002); Beck et al. (1996); Biederman et al. (2006); Chang et al. (2014); Cox et al. (2000); Fischer et al. (2007); Fried et al. (2006); Jerome et al. (2006); Koisaari et al. (2015); Murphy and Barkley (1996); NadaRaja et al. (1997); Narad et al. (2015); Richards et al. (2002, 2006); Safiri et al. (2013); Sobanski et al. (2008); Swenson et al. (2004); Thompson et al. (2007); Weiss et al. (1979); Woodward et al. (2000)

^b^Based on Barkley et al. (1996, 2002, 2005, 2006, 2007); Biederman et al. (2007, 2012a); Classen et al. (2013); Cox et al. (2000, 2004b, 2006a, b); Fischer et al. (2007); Groom et al. (2015); Kay et al. (2009); Knouse et al. (2005); Michaelis et al. (2012), Monahan et al. (2013); Narad et al. (2013); Oliver et al. (2012); Reimer et al. (2007, 2010); Weafer et al. (2008)

^c^Based on Cox et al. (2004a, 2008a, b, 2012); Fabiano et al. (2011); Fischer et al. (2007); Markham et al. (2013); Merkel et al. (2013); Sobanski et al. (2013); Verster and Cox (2008); Verster et al. (2008)

While differences between individuals with ADHD and those without appear obvious and have been replicated repeatedly, one has to consider that self- and informants’ reports as well as official records represent relative unreliable sources of data. For example, adolescents and adults with ADHD have been shown to have difficulties in self-reflection and self-evaluation and are therefore prone to under-report the severity of their symptoms (so-called positive illusory bias) as compared to informant reports or scores obtained by clinicians (Barkley and Cox 2007; Kooij et al. 2008; Manor et al. 2012; Smith et al. 2000; Zucker et al. 2002). One can assume that this proneness to minimise deficits and risks holds true for driving as well, as adults with ADHD have been observed to overestimate their driving performance (Knouse et al. 2005) which might indicate a lack of awareness of the severity of their driving problems. While this tendency makes conclusions about the self-reported driving behaviour of individuals with ADHD in general more conservative (because the driving deficits and risks might actually be higher than reported) (Barkley and Cox 2007), there are other problems related to self-, informant- and official reports which might result in a more serious underestimation of the actual problem. From the literature on fitness to drive of patients with acquired brain lesions or neurodegenerative conditions, it is known that patients who have some awareness of their driving problems often modify their behaviour to adapt to their difficulties. For example, patientcrashes, speeding, and driving errors might drive less or avoid driving at times and in situations requiring an increased cognitive effort such as driving at busy times or at night, on busy roads or in adverse weather conditions (Rizzo and Kellison 2010). This might apply to individuals with ADHD as well, since they also show some awareness of their driving problems (as reflected by their self-reports). The findings of Safiri and colleagues (2013) provide some support for this assumption. In a case–control study, these authors examined the association between adult ADHD and motorcycle traffic injuries and found that participants who were involved in motorcycle traffic injuries did not only show higher ADHD scores but also had less experience with driving at night. However, it has to be pointed out that Safiri et al. (2013) did not examine individuals who were formally diagnosed with ADHD. In contrast, Vaa (2014) mentioned in his review that drivers with ADHD appear to drive more than drivers without the condition, which would mean that self-, informant- and official reports may tend to overestimate the driving difficulties of individuals with ADHD.

Information derived from official driving records, such as crash records are particularly difficult to interpret. These records usually disregard the multifactorial character of many crashes and are often incomplete with minor crashes frequently not being reported, depending on the country. For example, a recent study that included the records of an impressive sample of more than 17.000 patients with ADHD (Chang et al. 2014) in Sweden could only analyse ‘serious transport accidents’ as defined as an emergency hospital visit or death due to transport accident, because less severe accidents have not been registered in the records analysed. Furthermore, official records are usually limited to specific aspects of conduct (i.e., traffic offences) or driving outcomes (e.g., accidents), neglecting other possible indicators of unsafe driving. Taking the limitations of self- and official reports into consideration, it is not surprising that the relationship between official driving records and patients’ self-reports is only moderate for both the frequency of accidents and frequency of traffic citations. In fact, the correlation coefficients range between *r* = 0.39 and *r* = 0.41 (Barkley et al. 2002; Barkley and Cox 2007), indicating that official records and self-reports of individuals with ADHD share only 15–17 % of their variance.

Another issue is that the selection of variables within studies can have quite an impact on the outcome of studies. For example, the (mean) number of road accidents in which samples of patients with ADHD are involved in represents a highly relevant measure for the driving risk related with ADHD. Several studies have provided clear evidence that the (mean) number of road accidents increases in individuals with ADHD (e.g., Barkley et al. 1993, 1996, 2002; Richards et al. 2002; Weiss et al. 1979). However, if the proportion of individuals with ADHD who are involved in road accidents is compared to the proportion of individuals without the condition, no significant increase has been observed (e.g., Barkley et al. 1996, 2002; Fischer et al. 2007), indicating that a sub-group of individuals with ADHD may account for a disproportionate number of accidents, instead of ADHD being generally related with increased driving difficulties. This assumption is supported by Barkley and colleagues (1993) who failed to find any differences between the proportions of individuals with ADHD and individuals without the condition who experienced at least one road accident, but could reveal a difference between these groups for those who had multiple road accidents (Barkley et al. 1993). Furthermore, in an internet survey, 60 % of drivers with ADHD reported no (minor) collisions within the previous 12 months (Cox et al. 2011b). Moreover, Chang and colleagues (2014) demonstrated that the increased number of serious road accidents in individuals with ADHD is experienced by a small proportion of individuals with ADHD. These authors reported that 6.5 % of men and 3.9 % of women with ADHD experienced road accidents during the 4-year follow-up period of the study in comparison to 2.6 % of men and 1.8 % of women without ADHD. In this context, it also appears relevant to point out that an increase in the number of road accidents of individuals with ADHD were not found across all studies.

#### Driving simulators

Assessments with driving simulators have good face validity with regard to the measurement of driving skills. They are a safe way of assessing driving behaviour in potentially dangerous situations by avoiding the risks associated with on-road testing. Moreover, they are considered reliable assessment tools since an exact replication of the conditions under which an individual’s driving is assessed (e.g., concerning weather, traffic, visibility, time of day) is possible across participants (Rizzo and Kellison 2010). However, despite an association has been found between simulator and on-road behaviour (e.g., De Waard and Brookhuis 1997; Kaptein et al. 1996), it has to be considered that assessments with driving simulators often lack in ecological validity (Vaa 2014). For example, there is no one-to-one relationship between accidents during a drive in a driving simulator and real on-road driving performance. In addition, driving simulations measure short-term driving skills instead of the individually established habits and practices. This might explain why the skills assessed with driving simulators do not necessarily perfectly predict on-road driving behaviour (Lew et al. 2005; Lundqvist et al. 2000). Furthermore, there are usually no clear standards yet with regard to the driving scenarios (e.g., traffic, weather) and the duration of simulator test drives. For example, short drives of only a few minutes might just not contain sufficient critical events (e.g., unexpected braking of a lead vehicle or unexpected merging of a car into the driver’s lane) to reveal driving difficulties, such as an increased number of collisions (Knouse et al. 2005). So, since driving scenarios applied across studies can vary significantly, they may not be sensitive to the deficits shown by individuals with ADHD or other conditions. Simple driving simulation systems, in particular, may be unsuited to detect subtle difficulties (Barkley et al. 2002). More advanced driving simulators, however, are expensive (Schultheis et al. 2007) and too rarely available to allow routine assessments of driving abilities of individuals with ADHD outside the research context.

In accordance with studies analysing self-, informant- and official reports, studies using driving simulators have also revealed that individuals with ADHD display various driving difficulties including an increased rate of collisions and crashes, speeding, and driving errors (Barkley et al. 1996, 2002, 2005, 2006; Barkley and Cox 2007; Biederman et al. 2007a, b, Classen et al. 2013; Cox et al. 2000, 2004b, 2006b; Fischer et al. 2007; Groom et al. 2015; Kay et al. 2009; Knouse et al. 2005; Michaelis et al. 2012; Monahan et al. 2013; Narad et al. 2013; Oliver et al. 2012; Reimer et al. 2007, 2010; Weafer et al. 2008). Furthermore, poorer steering control and increased lane swerving have been observed in both adolescents and adults with ADHD (Table 1). These differences should not been understood as being independent from each other. For example, increased speeding and poor steering control likely increase the risk of collisions.

#### On-road driving

Studies examining driving behaviour during on-road driving corroborate the findings presented in the previous two sections (Cox et al. 2004b, 2008b, 2012; Fabiano et al. 2011; Fischer et al. 2007; Markham et al. 2013; Merkel et al. 2013; Sobanski et al. 2013; Verster and Cox 2008). In comparison to individuals without ADHD, adolescents and adults with ADHD showed an increased number of collisions, speeding, more hard braking and sudden deceleration, more weaving of their car, as well as more driving errors in general (see Table 1). These findings are particularly important because on-road driving evaluation is considered to represent the gold standard in the assessment of driving abilities (Rizzo and Kellison 2010). However, on-road driving evaluation is associated with certain risks, because individuals with ADHD are actually interacting in a real traffic environment. Accident risk may even be increased during on-road driving evaluations, as individuals are on the one hand aware of the importance of the evaluation and on the other hand aware that they are being observed. This might increase tension and distraction during driving in some individuals. Since real traffic cannot be controlled (in contrast to the traffic scenarios used in driving simulation), traffic situations may also vary considerably between car rides. Furthermore, on-road driving evaluation is expensive and might require that patients take over the costs incurred. Finally, on-road driving evaluation is also only performed by a limited number of centres which might result in prolonged waiting times for patients and an increase in costs (i.e., expenses for travelling).

In conclusion, a number of studies have applied different methodological approaches to the assessment of driving abilities of individuals with ADHD. All approaches have significant strengths and weaknesses. Therefore, a clear conclusion concerning the question whether individuals with ADHD have indeed an increased driving risk can only be made when considering all studies and approaches in an integral manner. When the results of studies on driving in ADHD are pooled, one can only conclude that ADHD is related with increased driving difficulties and unsafe driving behaviour. However, it appears that not the entire group of individuals with ADHD is affected in a uniform fashion, but that there is rather a subgroup of drivers who are over involved in accidents (Barkley et al. 1993; Chang et al. 2014; Cox et al. 2011b). This group is presumably larger than the results of Chang and colleagues (2014) may imply (6.5 % of men and 3.9 % of women with ADHD experienced road accidents during a 4-year follow-up), because the files analysed by these authors only allowed the analysis of ‘serious transport accidents’. Less severe accidents, i.e., accidents that do not lead to death or emergency hospital visits, could not be considered. An important aim of future research should be a further examination of a wider source of data to confirm these findings. In this context, cognitive dysfunctions related to driving risks of individuals with ADHD might be of interest.

### What cognitive (dys)functions of individuals with ADHD are related to unsafe driving behaviour?

As already mentioned, adults with ADHD suffer from various cognitive deficits affecting various domains, including attention, executive functions, memory, language skills and spatial abilities (Hervey et al. 2004; Lange et al. 2010; Schoechlin and Engel 2005; Stefanatos and Wasserstein 2001; Woods et al. 2002). The majority of these deficits may also impair driving behaviour and driving performance (Lincoln and Radford 2013). In particular, in a number of studies it was found that inattention and distractibility, impulsivity, and reduced cognitive flexibility affect driving behaviour of individuals with ADHD adversely (Barkley et al. 2002; Fischer et al. 2007; Fried et al. 2006; Garner et al. 2012; Jerome et al. 2006; Merkel et al. 2013; Narad et al. 2013; Rosenbloom and Wultz 2011). Examples of consequences of inattention and increased distractibility with regard to driving may include late detection of critical situations and an increase in near-crashes/crashes, while increased impulsivity may result in unsafe manoeuvres and speeding. Cognitive flexibility is relevant when shifting the focus of attention among relevant stimuli and tasks. The integrity of cognitive flexibility is crucial for driving, because driving requires that many different tasks have to be performed at the same time or in quick succession at different levels of attention (Michon, 1985). For example, a typical driving situation may demand the monitoring of changing road conditions and the tracking of changing locations of neighbouring vehicles, as well as reading traffic signs and traffic lights (e.g., Kantowitz 2001; Owsley et al. 1991). If the focus of attention is not disengaged in time, for instance from the following traffic when checking the mirrors, other road users might be overlooked and the risk for accidents is increased. Inattention/distractibility, impulsivity, and cognitive flexibility consequently appear to form the basis on which screening measures should be developed to identify poor drivers with ADHD. However, because inattention/distractibility and impulsivity represent impairments which in part define ADHD, it remains unclear whether test measures can be developed that not only allow a distinction between low and high risk drivers with ADHD on a group level (e.g., Fried et al. 2006) but also on an individual level. To support the identification of high risk drivers with ADHD, several questionnaires have been proposed and applied in the assessment of individuals with ADHD, including the *Driving Behaviour Rating Scale* (e.g., Barkley et al. 1993, 1996, 2002; Fischer et al. 2007), the Driving Behaviour Questionnaire (e.g., Fried et al. 2006; Reimer et al. 2005; Woodward et al. 2000), the Survey of Driving (e.g., Richards et al. 2002), and the Jerome Driving Questionnaire (e.g., Jerome et al. 2006). Again, group effects could be demonstrated with these measures; however, there are no clear and generally accepted rules or criteria for a decision in an individual case. These questionnaires are easy and efficient to administer but limited due to their self-report character. Future research on driving abilities and risks of individuals with ADHD might benefit from advances made in the assessment of the fitness to drive of patients with neurological conditions. In this regard, a reduced contrast sensitivity or a poor Useful Field of View (UFOV) has been shown useful to predict driving performance of both healthy drivers and drivers with neurological conditions (Clay et al. 2005; George and Crotty 2010; Owsley et al. 1998). UFOV is a concept that can be defined as the area in which visual information can be acquired and processed without eye and head movement (Ball et al. 1998). The UFOV test requires both identification and localization of targets and can be described as a composite measure combining speed of processing, divided attention, and susceptibility to distraction. Moreover, it was demonstrated that higher-order cognitive functions as well as visual sensory functions are required for successful UFOV test performance (Clay et al. 2005; Owsley et al., 1995). Three subtests can be distinguished in the UFOV test. In the first subtest, the identification of a centrally presented target is required. The second subtest requires the identification of the central target along with the simultaneous localization of a peripheral target (measure of divided attention). The third subtest of the UFOV is similar to the first and second subtest, but additional includes visual distractors (Clay et al. 2005). As such, the UFOV appears quite interesting and could be suited for the assessment of driving-related cognitive abilities of individuals with ADHD. Differences between teenagers with ADHD and teenagers without the condition in single measures of the UFOV have already been reported lately (Classen et al. 2013). However, even though the UFOV is a promising mean for the prediction of driving safety, it has also been noted in the context of older drivers that UFOV alone will not explain the entire variance in driving performance (Bedard et al. 2008; Langford 2008). Furthermore, recent research has provided evidence of impaired visual acuity and visual fields in individuals with ADHD and demonstrated that visual problems may have an impact on driving performance of adults with ADHD (Classen et al. 2013; Kim et al. 2014; Martin et al. 2008). Since impairments of attention (e.g., distractibility) and executive functions (reduced cognitive flexibility and impulse control) as well as of visual acuity and visual field affect driving behaviour of adults with ADHD adversely, and because these functions have been found to be significantly improved following pharmacological treatment (e.g., Martin et al. 2008; Tucha et al. 2006b), the question arising from these findings is whether pharmacological treatment has the potential to improve driving behaviour of adolescents and adults with ADHD.

### What are the effects of pharmacological treatment on driving behaviour of individuals with ADHD?

Several studies have addressed the question of whether pharmacological treatments affect driving abilities of individuals with ADHD (Barkley et al. 2005, 2007; Biederman et al. 2012a, b; Chang et al. 2014; Cox et al. 2000, 2004b, 2006b, 2008b, 2012; Jerome and Segal 2001; Kay et al. 2009; Ludolph et al. 2009; Mikami et al. 2009; Sobanski et al. 2008, 2013; Verster and Cox 2008; Verster et al. 2008). In this respect, Gobbo and Louza (2014) performed recently a systematic review of the literature in which they summarised elegantly the pharmacological treatments effects on driving behaviour of individuals with ADHD. These studies go across the complete range of assessment approaches, including self- and spousal reports, official driving records, simulator driving, and on-road driving assessment and revealed positive effects of immediate and sustained release methylphenidate on various measures of driving performance, including frequencies of collisions and speeding. The effect sizes of improvements ranged between 0.2 and 1.3 (Cohen’s d). Beneficial effects on the driving of individuals with ADHD have also been reported for stimulants other than methylphenidate, such as long-acting mixed amphetamine salts and lisdexamfetamine dimesylate. While there is no information about the robustness of the effects of lisdexamfetamine dimesylate, the effects of long-acting mixed amphetamine salts appear less robust than those found for methylphenidate. However, despite the positive effects of long-acting mixed amphetamine salts, there is also some evidence of an increased number of on-road inattentive driving errors 16–17 h post-ingestion, suggesting possible rebound effects (Cox et al. 2008b). Also non-stimulant medication (atomoxetine), also used for the treatment of ADHD, has been explored with regard to the effects on driving abilities in individuals with ADHD. These studies yielded however conflicting results as two studies produced negative findings (Barkley et al. 2007; Kay et al. 2009) and only one study revealed improvements in driving behaviour of adults with ADHD treated with atomoxetine compared to a wait-list control group (Sobanski et al. 2013). Several factors were discussed that may have contributed to these conflicting results, such as differences in sample size, driving experience of patients, administered dose of atomoxetine, and the time between pharmacological treatment initiation and driving behaviour assessment. Chang and colleagues (2014) who examined the association between ADHD medication and serious transport accidents in a population-based study in Sweden (i.e., analysis of official driving records) also reported a reduced rate of accidents in men with ADHD when they were on medication as compared to periods without medication (stimulants or non-stimulants). On the basis of their data and the specific conditions in Sweden, these authors assumed that 41–49 % of serious accidents of males with ADHD might have been avoidable if they would have been on medication at the time of the accident. No differences between medication and non-medication periods were revealed for women with ADHD. In fact, a within-subject analysis even suggested an increase in the risk of traffic accidents for women with ADHD during medication periods. Chang and colleagues (2014) assumed that this finding was presumably a chance finding; however, this might need further clarification and should possibly also been understood in the context of the detrimental effects of long-acting mixed amphetamine salts as mentioned above (Cox et al. 2008b). Taken together, there is clear evidence that pharmacological treatment of ADHD improves driving performance significantly which may translate into a reduction of accident involvement and unsafe behaviour (Chang et al. 2014; Kuepper et al. 2012).

However, the value of the results of studies on the effects of pharmacological treatments is limited because of various methodological issues, including small sample sizes with the majority of studies (in particular the experimental studies) including samples of 30 or less participants. Moreover, a biased selection of participants often resulted in the recruitment of individuals with ADHD with a recent history of driving mishaps and a predominance of male young participants. These are relevant issues, since studies on older participants and larger samples found fewer and weaker positive effects of pharmacological treatment on driving performance (Jerome et al. 2006). Further methodological weaknesses of several studies comprise a complete lack of or, at least, unclear randomisation procedures, failure to assess medication adherence, absence of placebo groups or placebo conditions and the absence of control groups without ADHD. The latter point is crucial, since the question whether pharmacological treatment normalises driving behaviour is paramount and has many implications for patients, clinicians, and policy makers. Relevant questions, for example, might be whether clinicians should recommend pharmacological treatment to adult drivers with ADHD (e.g., when weighing the benefits of pharmacological treatment against possible side effects) or whether pharmacological treatment should even become a requirement for adults with ADHD to drive a vehicle? Previous research has demonstrated that pharmacological treatment may result in behavioural and cognitive improvements but might not necessarily normalise behaviour (Gualtieri and Johnson 2008, 2011a; Tucha et al. 2006b; Whalen et al. 2006). This means that even if individuals with ADHD respond positively to pharmacological treatment and show considerable improvements (e.g., in cognition), they are often still impaired while on medication when compared to healthy control groups. Thorough analysis of the data of one of the few studies considering a control group (Cox et al. 2000) indicate that stimulant medication indeed does improve driving performance but does not normalise performance. In this context, it is important to emphasise that many of the results mentioned in this chapter were based on driving simulation studies. If a driving simulator is understood as a complex attention test, it appears not so surprising that performance is improved following pharmacological treatment, since a wealth of studies on children and adults with ADHD already demonstrated positive effects of pharmacological treatment on attention functions as assessed with neuropsychological test procedures (e.g., Tucha et al. 2006a, b, 2011b). Generalisation of these treatment effects into daily life, however, is still elusive and might represent a particular problem with regard to driving, because not all abilities assessed in driving simulators may correspond to on-road driving (Rizzo and Kellison 2010). Therefore, despite all previously reported improvements of driving abilities observed following pharmacological treatment, additional research is necessary to address the methodological problems of the available literature and to clarify whether the observed medication-induced improvements really translate to real life driving. Furthermore, it would be interesting to know whether the beneficial medication-induced effects on driving are stable over time. In this regard, non-pharmacological treatment approaches should also be researched in more detail. So far, only a limited number of studies examined the effects of behavioural interventions on driving related behaviours (Cox et al. 2006b; Fabiano et al. 2011; Markham et al. 2013; Poulsen et al. 2010). In these studies, unfortunately only small samples (*n* < 10) were included but nevertheless it was found that in comparison to automatic transmission, manual transmission increases arousal and driving safety (as assessed by self-reports and simulated driving) (Cox et al. 2006b), that hazard perception training improved response times in a video-based hazard perception test (Poulsen et al. 2010), and that behaviour modification programmes using incentives and disincentives have the potential to reduce speeding behaviour (on-road driving) (Markham et al. 2013). In addition, some promising effects on on-road driving were demonstrated for the Supporting a Teen’s Effective Entry to the Roadway (STEER) programme, which is a multicomponent intervention integrating driving targeted behavioural parent training, communication training, driving practice on a simulator for teens with ADHD, and parental monitoring of on-road driving (Fabiano et al. 2011).

## General conclusion

There is convincing evidence that adolescents and adults with ADHD have different and adverse driving outcomes than individuals without the condition. An increased number of accidents and speeding violations appear to be the most robust driving difficulties in ADHD. A recent meta-analysis estimated that the overall relative risk of accidents for drivers with ADHD is 1.36 (uncontrolled for mileage) or 1.23 when controlled for mileage (Vaa 2014). However, it appears that this increased risk might not affect all individuals with ADHD in a uniform manner. In this context, previous comorbid disorders (e.g., oppositional defiant disorder or conduct disorder in childhood or adolescence) might also be of interest, as comorbidity seems to contribute to road accidents in individuals with ADHD (Vaa 2014). Further research is necessary on this topic and one particular question is whether at risk drivers can be identified, e.g., by neuropsychological testing or by questionnaires designed to measure driving behaviour. There are already several questionnaires available; however, further validation in particular with regard to ADHD is necessary. Despite various cognitive functions being related with driving difficulties (e.g., inattention and impulsivity), these functions may not be helpful in detecting high risk drivers with ADHD or in predicting driving outcome of individuals with ADHD, since impairments in these functions are defining criteria for the diagnoses of ADHD. Other functions and measures, therefore, have to be identified. In this regard, researchers might find inspiration in the findings of studies performed in the field of fitness to drive of patients with neurological conditions (e.g., reduced contrast sensitivity or poor UFOV). Pharmacological treatment of ADHD, in particular stimulant drug treatment, appears to be beneficial regarding driving difficulties experienced by individuals with ADHD. However, previous studies have numerous methodological weaknesses that should be taken into account. Furthermore, more research on non-pharmacological interventions is desirable. This research should be based on the findings made in the field of traffic psychology.
